# Exploring and Deepening the Facets of Mentalizing: The Integration of Network and Factorial Analysis Approaches to Verify the Psychometric Properties of the Multidimensional Mentalizing Questionnaire (MMQ)

**DOI:** 10.3390/ijerph20064744

**Published:** 2023-03-08

**Authors:** Alessio Gori, Eleonora Topino

**Affiliations:** 1 Department of Health Sciences, University of Florence, Via di San Salvi 12, Pad. 26, 50135 Florence, Italy; 2 Integrated Psychodynamic Psychotherapy Institute (IPPI), Via Ricasoli 32, 50122 Florence, Italy; 3 Department of Human Sciences, LUMSA University of Rome, Via della Traspontina 21, 00193 Rome, Italy

**Keywords:** mentalization, multidimensionality, multilevel model, factor analysis approach, network analysis approach, self-report measure, MMQ

## Abstract

Mentalization is a complex and multifaceted trans-theoretical and trans-diagnostic construct that has found increasing application in the clinical context. This research aimed at deepening the psychometric properties of the Multidimensional Mentalizing Questionnaire (MMQ), a 33-item theoretically based self-report questionnaire allowing for a comprehensive assessment of mentalizing, by integrating factor analysis and network analysis approaches. A sample of 1640 participants (*M_age_* = 33 years; *SD* = 13.28) was involved in the research. The six-factor structure was confirmed for the MMQ, and both the total and the subdimensions demonstrated good reliability. The network analysis has further enriched these results, showing the central role of the items attributable to Emotional Dysregulation or Reflexivity in influencing the network as well as the contribution of aspects related to Relational Discomfort in managing the flow of communication flow. Such findings may have useful clinical implications and emphasize the usefulness of the MMQ in both research and clinical practice.

## 1. Introduction

The concept of mentalization refers to a higher-order skill that enables individuals to understand themselves and others in terms of subjective states and mental processes [1], carefully defined by Bateman and Fonagy as “*the mental process by which an individual implicitly and explicitly interprets the actions of himself and others as meaningful on the basis of intentional mental states such as personal desires*, *needs*, *feelings*, *beliefs*, *and reasons*” (p. 21) [2]. It is a transtheoretical and transdiagnostic construct that allows the meeting of currents of thought from developmental psychology, psychoanalysis, and cognitive neuroscience to understand aspects of individual functioning [3]. In this regard, mentalizing was positively associated with self-esteem, general, social, and role functioning [4], better adaptation to stress [5], positive coping strategies [6], and resilience [7,8]. In a prospective longitudinal study on a non-clinical population of adolescents, it was found to be predictive of well-being after 8 years [9]. On the other hand, a considerable line of research has also highlighted alterations in mentalizing processes in a wide range of psychopathological conditions (see Ballespí et al. [10]; Luyten et al. [11] for reviews): eating disorders [12], depressive disorders [13,14,15], anxiety disorders [16], somatoform disorders [17], addictions [18,19], psychotic disorders [20], trauma-related disorders [21,22], and personality disorders [23,24], to name a few. In this framework, although important advances in research in the field have occurred thanks to the use of valid scales for the mono-dimensional assessment of mentalization, such as the Reflective Functioning Scale (RFS) [25], some evidence suggests that different diseases may have different forms of imbalances in mentalizing, involving its dimensions in specific ways [26,27]. Therefore, given its potential application in the treatment of psychopathology (see Malda-Castillo et al. [28] for a review), both research and clinical practice could benefit from tools that manage to put a multidimensional lens on mentalizing and evaluate problems related to its components to favour targeted and specific interventions. In this light, the Multidimensional Mentalizing Questionnaire (MMQ) [29] seems to be a promising measure.

### 1.1. The Multidimensional Mentalizing Questionnaire (MMQ): A Theoretically Based Measure for the Assessment of a Multifaceted Construct

The *Multidimensional Mentalizing Questionnaire* (MMQ) is a 33-item self-report questionnaire that allows for a comprehensive assessment of mentalizing [29]. The measure was developed considering the multifaceted nature of the construct, conceptualized as a process involving four axes [30]:

- *Automatic—Controlled mentalizing.* Automatic (or implicit) mentalizing refers to operations of rapid processing requiring little or no effort, awareness, or intention [31]. It is the prevalent configuration in daily life and in normal social interactions that do not require higher levels of attention [32]. However, more complex demands for communication and collaboration may be the source of a shift to controlled (or explicit) mentalizing, which reflects slower processing operations and requires greater levels of effort, awareness, and intention: it is more reflective, conscious, and deliberate [31,33,34].
- *Self—Other mentalizing*. This axis describes two closely and ontogenetically intertwined dimensions: mentalizing the self refers to the ability to reflect on one’s own lived and emotional and physical experiences, while mentalizing about others is characterized by a focus on other people and refers to the ability to correctly understand the reasons behind the behavior of others [33,35].
- *Internal—External mentalizing*. Mentalization can imply making inferences based on internal or external indicators. Therefore, internal mentalizing is a process focused on the internal world (e.g., thoughts, beliefs, desires, feelings, emotions, etc.). On the other hand, external mentalizing focuses on the characteristics and external manifestations of mental states (e.g., prosody, body posture, facial expressions, etc.) [33,36].
- *Cognitive—Affective mentalizing*. Cognitive mentalization finds its precursor in the concept of theory of mind [37] and describes the process of “thinking about thinking”, i.e., the ability to name, recognize, and reason about mental states [30]. Affective mentalization, on the other hand, implies the ability to understand the emotional dimension of mental states, and represents a necessary element for any genuine experience of empathy [30,38].

Good mentalizing ability is conceptualized as the result of a balance between these polarities that favours a flexible use of each dimension according to needs [39], while mentalizing difficulties are the result of imbalances, poor integration, or excessive polarization in the different axes [40].

**Figure 1:**
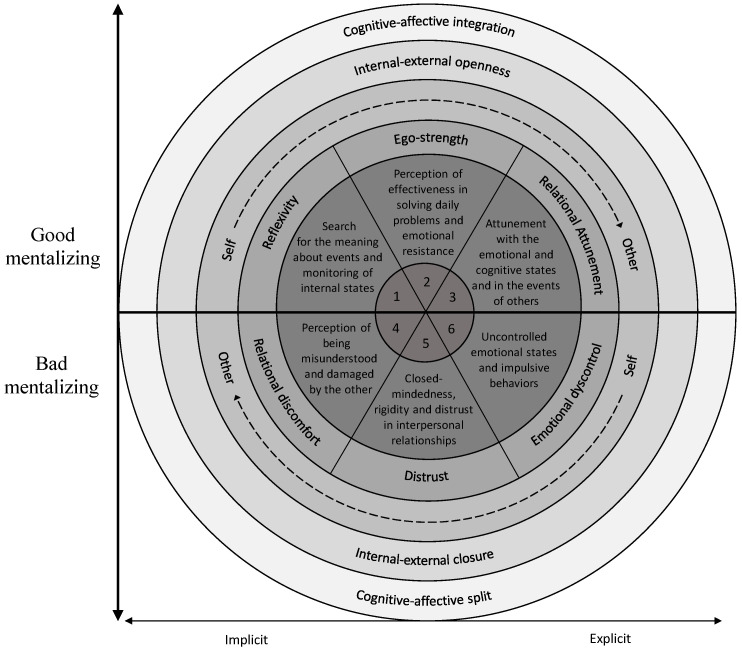
The integrated and multilevel model of mentalizing. Note: Reproduced from Gori and colleagues [29].

Based on this framework, the MMQ [29] allows for the evaluation of both the global level of mentalizing and four sub-components of the construct (see Figure 1), three of which are positive (conceptualized as expressions of integration of the dimensions of the four axes) and three are negative, theoretically opposite to the first ones (conceptualized as expressions of imbalances and extremes of the dimensions of the four axes):

(1) *Reflexivity*. This positive dimension refers to a propensity to understand the profound meaning of one’s life events, characterized by curiosity and the desire to analyze one’s experiences.
(2) *Ego-strength*. This positive dimension refers to the ability to effectively manage daily difficulties and painful experiences, characterized by a sense of efficacy and realistic confidence.
(3) *Relational Attunement*. This positive dimension refers to the inclination to tune into the emotional and cognitive states of others, deeply understanding their experiences.
(4) *Relational Discomfort*. This negative dimension refers to experiences of discomfort and difficulty in the interpersonal sphere, linked to the perception of being misunderstood and damaged by others.
(5) *Distrust*. This negative dimension refers to a closed-minded disposition towards the outside world, rigidity, and distrust in interpersonal relationships.
(6) *Emotional Dyscontrol*. This negative dimension refers to a tendency towards impulsivity, accompanied by difficulty in managing and processing the emotional components of one’s experience.

The questionnaire has shown good indications of internal consistency and construct validity. It also demonstrates clinical sensitivity by showing significant differences between a community sample and subjects with different diagnoses [29]. Given these properties, the MMQ appears to be a particularly functional questionnaire that may be used to facilitate the understanding of the mentalizing process both in research and clinical practice.

### 1.2. Aims of the Study

The construct of mentalizing appears complex and multifaceted, encompassing a series of distinguishable dimensions, albeit in interaction with each other [30,41]. The effectiveness found in the promotion of medical and psychological treatments [42,43,44,45] suggests the possibility that this function may be a key element for healthy individual functioning. Therefore, in light of the fruitful clinical applications, the use of updated and solid instruments appears to be of high importance to allow for a multidimensional evaluation of the construct.

Given this framework, the general objectives of the present research are to provide a greater comprehension of a multilevel evaluation of mentalizing and, at the same time, further enrich the psychometric evidence of the Multidimensional Mentalizing Questionnaire (MMQ) [29] in a large community sample to facilitate its use in various contexts. Indeed, in the validation study, preliminary results about the psychometric properties of the scale were provided [29], and both the clinical and research fields would benefit from further details and directions about the interpretation of the scores.

Therefore, this study specifically aims at:

- Confirming the factor structure of the MMQ based on the Factor Analysis Approach to support the theoretically sound base of the questionnaire;
- Establishing the cut-off points for both the MMQ total score and its subdimensions to allow clinicians to address specific aspects towards which to direct clinical and preventive activity;
- Deepening the statistical characteristics of the MMQ by using a novel Network Analysis approach to assess the connection between items and their centrality properties.

## 2. Materials and Methods

### 2.1. Participants and Procedure

A sample of 1640 participants was involved in this research (see Table 1). Their mean age was 33 years (*SD* = 13.28) and they were predominantly women (76%), single (66%), students (33%), and had a university degree (31%). The recruitment process was implemented online through a snowball sampling technique, and participation in this research was voluntary and anonymous. The administration took place online on the Google Form platform, where the survey was preceded by an information page. Before starting, each participant declared to be sufficiently informed about the data processing methods and general objective of the research and provided their informed consent electronically. All the procedures of this research were approved by the first author’s institutional Ethical Committee.

### 2.2. Measure

The *Multidimensional Mentalizing Questionnaire* (MMQ) is a self-report measure developed by Gori and colleagues [29], which allows for an assessment of the levels of mentalization based on the construct conceptualization covering four different axes: (1) cognitive–affective; (2) self–other; (3) outside–inside; and (4) explicit–implicit [30]. The questionnaire consists of 33 items (see Appendix A), rated on a five-point Likert scale from 1 (Not at all) to 5 (A great deal), and allows for the measurement of six sub-dimensions, three of which are positive (*Reflexivity*, *Ego-strength*, and *Relational Attunement*, characterized by a balance in the four axes) and three are negative (*Relational discomfort*, *Distrust*, and *Emotional Dyscontrol*, characterized by imbalances and maladaptive functioning along the four axes). The formers describe functional components of mentalizing and are opposite to the latter, which instead describe referred to failures and distortions (see Figure 1). Furthermore, the MMQ allows for a total score by summing all the items after reversing those of the negative subdimensions: higher scores indicate higher levels of mentalizing. Finally, the measure presented good psychometric properties in the first validation study, showing theoretically sound factor structure, good indications of divergent validity, and good internal consistency (MMQ total, *α* = 0.75; Reflexivity, *α* = 0.89; Ego-strength, *α* = 0.81; Relational Attunement, *α* = 0.82; Relational discomfort, *α* = 0.76; Distrust, *α* = 0.74; Emotional Dyscontrol, *α* = 0.72).

### 2.3. Data Analysis

Data were analyzed using the SPSS (v. 21.0; IBM, New York, NY, USA), AMOS (v. 24.0; IBM, New York, NY, USA), and JASP (v. 0.16.4; JASP Team, Amsterdam, The Netherlands) software for Windows. First, item analysis was conducted to test the normality of the data distribution: an absolute skew value equal to or less than 2 or an absolute kurtosis equal to or less than 7 has been considered indicative of normality [46]. Then, the Kaiser-Meyer-Olkin (KMO) statistic and Bartlett’s test of sphericity were performed: the KMO value of more than 0.7 and Bartlett’s test being statistically significant (*p* < 0.001) indicated the sampling adequacy for factor analysis [47]. Therefore, the statistical adequacy of the six-factor structure elaborated in the validation study [29] was tested by implementing a Confirmatory Factor Analysis (CFA) and considering the following indices: the chi-square (*χ*^2^) of the model, suggesting a good fit when the *p*-value is statistically non-significant [48]; the Normed-Fit Index (NFI), the Tucker Lewis index (TLI), and the Comparative Fit Index (CFI), suggesting a reasonable fit for values of 0.90 and higher [49,50]; the Root Mean Square Error of Approximation (RMSEA) and Standardized Root Mean Square Residual (SRMR), suggesting a reasonable fit for values less than 0.08 and a poor fit for values greater than 0.10 [51]. The factorial structure of the MMQ was also assessed by using the Δ*χ*^2^ to compare the six-factor model with alternative models [52,53], i.e., a higher-order model (with the “Positive Dimensions” and “Negative Dimensions” as second-order constructs) and the unifactorial solution: *p* < 0.05 supports a statistical difference in the fit of the models to the data [54]. Cronbach’s alpha [55] and McDonald’s omega [56] coefficients were calculated to evaluate the internal consistency of the measure. In addition, the heterotrait-monotrait ratio of correlations (HTMT) [57] was implemented to test the discriminant validity among the subscales by using an AMOS plugin [58], suggesting good discrimination for values < 0.85, although a threshold of <0.90 may be considered acceptable [57]. Furthermore, the cut-off points of both the MMQ total score and the subscales were calculated by determining the 25th and 75th percentiles for each scale to indicate low (<25th percentile), average (25th to <75th percentile), and high (>75th percentile) scores. Then, a network analysis was conducted to further investigate the MMQ’s internal structure, exploring the complex relational patterns of the items. The EBICglasso (Extended Bayesian Information Criterion Graphical Least Absolute Shrinkage and Selection Operator) with a Tuning Parameter of 0.5 was selected to estimate the network. A network is characterized by two core components: nodes (the observed variables, i.e., the questionnaire items in this research) and edges (indicating the magnitude of relationships between the nodes) [59]. Based on Ferguson’s [60] guidelines, edge weights less or equal to 0.2 were interpreted as small, edge weights more than 0.2 and less or equal to 0.5 were interpreted as moderate, and edge weights more than 0.5 were interpreted as large. Furthermore, three centrality indices of the network were explored: betweenness, closeness, and degree [61]. The *betweenness* indicates the frequency with which a specific node is located in the shortest distance between two other nodes; the *closeness* indicates the amount of short direct and indirect connections between this node and all the others in the network; the *degree* indicates the weighted number of edges of the node based on both the quantity and the strength of all its connections, and represents the overall influence of a node in the network [62]. Finally, the Bootstrap analysis with 5000 simulated samples (95% confidence intervals) was performed to examine the edge stability [62].

## 3. Results

### 3.1. Factor Structure and Internal Consistency

The item analysis suggested a normal distribution, with skewness values ranging from −1.39 (item 6) to +1.85 (item 9), and kurtosis values ranging from −1.24 (item 15) to 1.66 (item 6). The KMO index of 0.94 and the statistical significance of Bartlett’s test of sphericity (*p* < 0.001) supported the suitability of the data for factor analysis. 

**Figure 2:**
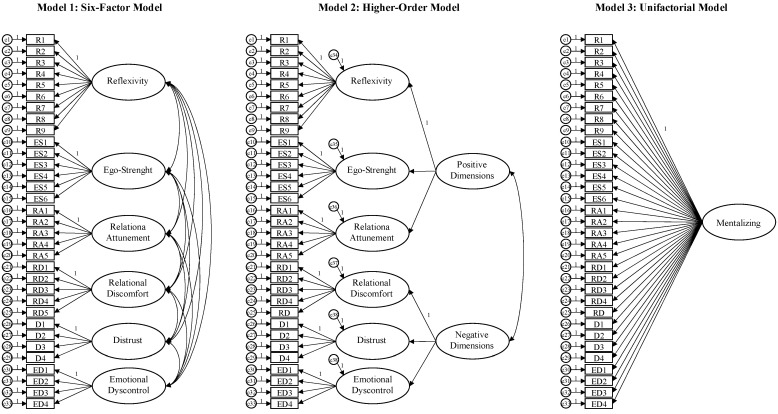
The tested factorial structure models for the MMQ.

The CFA confirmed the statistical adequacy of the 6-factor structure (see Table 2 and Figure 2): although the *χ*^2^ was statistically significant (*p* < 0.001), the other indices showed acceptable values: NFI = 0.90, TLI = 0.90, CFI = 0.91, RMSEA = 0.06, and SRMR = 0.06. Furthermore, the exploration of the chi-square variation further supported the fit superiority of the 6-factor structure compared with both the higher-order model (Δ*χ*^2^ = 307.46, Δ*df* = 8, *p* < 0.001) and the unifactorial model (Δ*χ*^2^ = 11,328.575, Δ*df* = 15, *p* < 0.001).

Concerning the internal consistency of the MMQ, Cronbach’s alpha and McDonald’s omega coefficients showed good values for both the total score (α = 0.90; ω = 0.88) and the subscales (see Table 3). Furthermore, the HTMT inference did not indicate problems with discriminant validity for the MMQ subscales, which showed associations below the threshold value of 0.85 (Table 3).

**Figure 3:**
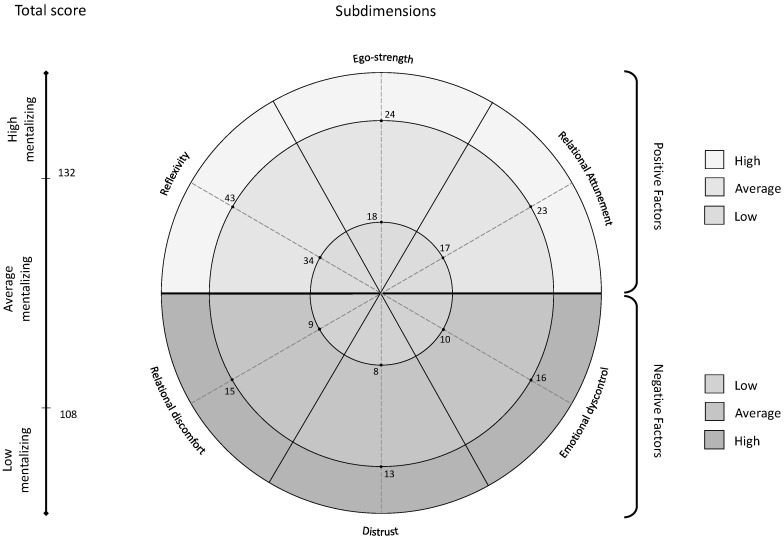
Scoring chart for the Multidimensional Mentalizing Questionnaire.

The cut-off points for the MMQ total score and each dimension were calculated based on the 25th and 75th percentiles, indicating low, average, and high scores (see Table 3 and Figure 3). Specifically, concerning the total score, MMQ scores less than 108 (25th percentile) indicate “Low mentalizing”; MMQ scores greater than or equal to 108 (25th percentile) and less than or equal to 132 (75th percentile) indicate “Medium mentalizing”; MMQ scores greater than 132 (75th percentile) indicate “High mentalizing” (see Table 3 and Figure 3).

### 3.2. Network Analysis

**Figure 4:**
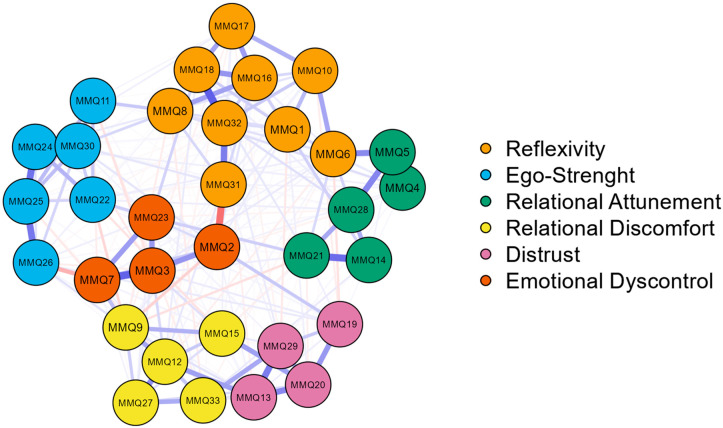
Gaussian graphical model for the Multidimensional Mentalizing Questionnaire: the network structure. Note: Red lines indicate negative associations between items; blue lines indicate positive associations between items; thicker lines indicate stronger edge weights.

The network of the 33 MMQ items is shown in Figure 4.

The analysis showed edges of moderate/high intensity between nodes attributable to the same factor (see Figure 4): items 6–10 (0.21), 8–16 (0.22), 16–17 (0.23), 16–18 (0.25), 17–18 (0.22) for Reflexivity; items 24–25 (0.33), 24–30 (0.39), 25–26 (0.32) for Ego-Strength; items 4–5 (0.59), 5–28 (0.29), 14–21 (0.34), 21–28 (0.21) for Relational Attunement; items 9–12 (0.21), 12–27 (0.27) for Relational Discomfort; items 13–20 (0.28), 13–29 (0.31), 19–20 (0.24), 20–29 (0.20) for Distrust; items 2–3 (0.25), 3–7 (0.36), 3–23 (0.24), 7–23 (0.23) for Emotional Dyscontrol. Some moderate and positive associations between items belonging to different positive factors, i.e., items 5–6 (0.28) relating Relational Attunement and Reflexivity, or negative ones, i.e., items 12–13 (0.23) and items 15–20 (0.22) relating to Relational Discomfort and Distrust in both pairs, were identified (see Figure 4). Finally, negative and moderate edge weight was found between two items attributable to two dimensions conceptualized as opposite (see Figure 4): items 2–31 (−0.34).

**Figure 5:**
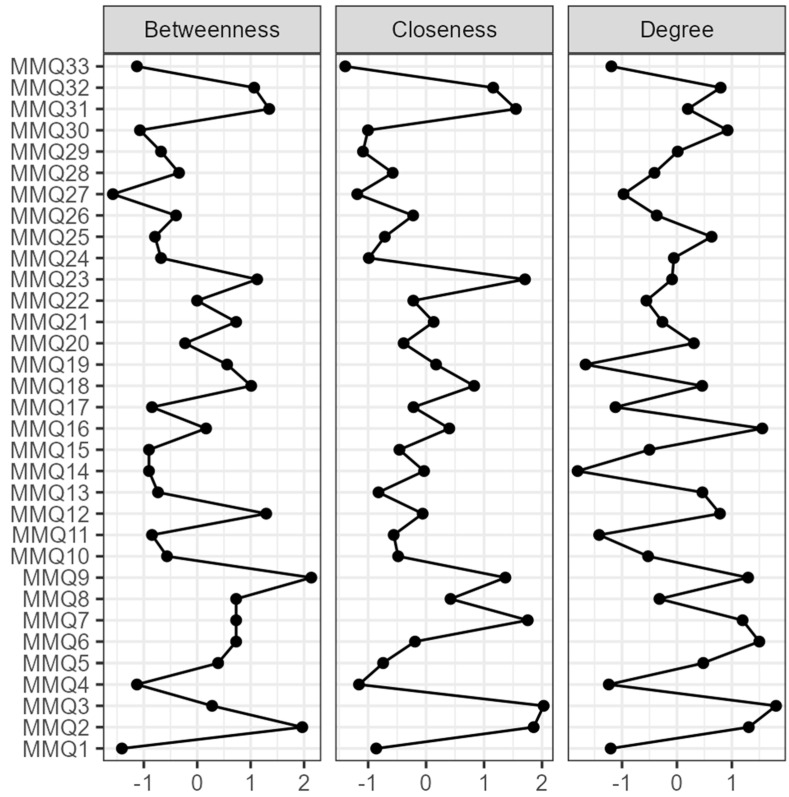
Centrality plot of the MMQ. For each index, higher values indicate greater centrality in the network.

Furthermore, the three centrality indices of the network (betweenness, closeness, and degree) were shown in Figure 5. Item 3 (“*I sometimes experience mood swings I can’t control*”) had the highest degree, followed by item 16 (“*I ponder over what happens to me*”), item 6 (“*Understanding what others feel is crucial in understanding their actions*”), and item 2 (“*I am an impulsive person*”); these items may be attributable to the opposite factors of Reflexivity or Emotional dysregulation and represented the nodes with the highest strength and overall influence in the network (see Table 4 and Figure 5).

Item 3 (“*I sometimes experience mood swings I can’t control*”) has also shown the highest Closeness, followed by item 2 (“*I am an impulsive person*”), items 7 (“*I sometimes feel like I am losing control of my emotions*”), item 23 (“*It happens to me to have conflicting emotions*”), and item 31 (“*I am a thoughtful person*”). These nodes, attributable to the dimensions of Reflexivity or Emotional dysregulation, are the ones that have higher direct and indirect connections with the other nodes in the network (see Table 4 and Figure 5).

Item 9 (“*Relationships with other people prevent me from being myself*”) was found to be the node with the highest Betweenness, followed by item 2 (“*I am an impulsive person*”), item 31 (“*I am a thoughtful person*”), and item 12 (“*Others don’t understand me*”). These items, attributable to the dimensions of Relational discomfort, Reflexivity or Emotional dysregulation, act as bridge connectors between other nodes and may therefore control the information flow of the network. 

**Figure 6:**
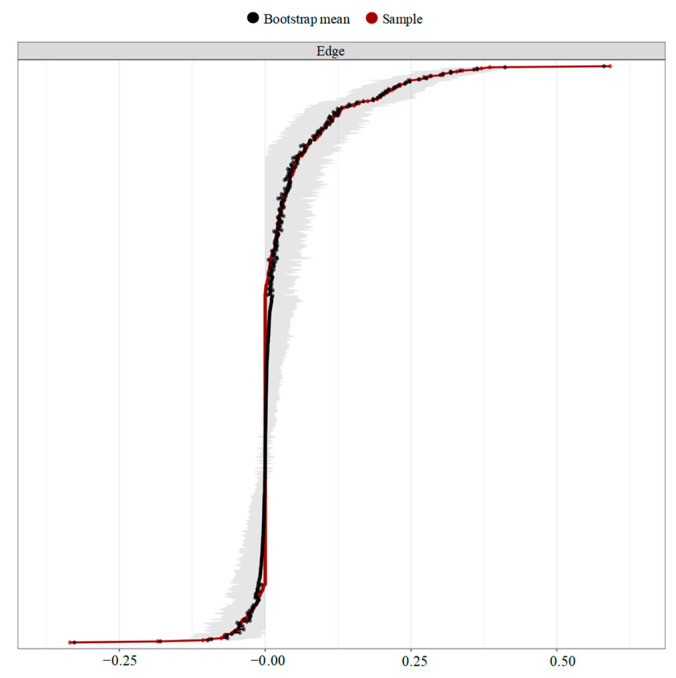
Bootstrap tests of the edge weight accuracy. Note: The grey areas represent the 95% confidence intervals.

Finally, the Bootstrap analysis indicated that the associations in the network have been estimated with acceptable accuracy (see Figure 6).

## 4. Discussion

The Multidimensional Mentalizing Questionnaire (MMQ) [29] is a 33-item self-report questionnaire that aims to offer an assessment of mentalizing through a multifocal lens that captures the complexity of the construct (see Figure 1). The present research aimed at enriching the exploration of its psychometric properties by integrating the Factor Analysis Approach with the novel Network Analysis Approach to further test the internal structure of the questionnaire and to assess the connection between items and their centrality properties.

### 4.1. Factor Analysis Approach

The confirmatory factor analyses (CFAs) supported the goodness of fit of the six-factor model for the MMQ (see Figure 1), echoing the original study [29]. As shown in Figure 1, the measure consists of three positive factors (*Reflexivity*, *Ego-Strength*, and *Relational Attunement*) and three negative ones (*Relational Discomfort*, *Distrust*, and *Emotional Dyscontrol*). The positive dimensions have been conceptualized to reflect balance in the polarities of the four axes involved in mentalizing (cognitive–affective; self–other; outside–inside; explicit–implicit) [30], consistent with previous evidence showing that aspects of meta-cognition, openness, and interpersonal attunement were positively associated with mentalization [63,64,65]. On the other hand, the negative dimensions have been theorized as opposite to the positive ones, and express poor integration between poles, imbalances, and therefore failures and distortions in mentalizing [30], in line with previous research highlighting that social withdrawal, interpersonal distrust, and emotional dysregulation were negatively associated with the levels of mentalizing [66,67,68]. Furthermore, Cronbach’s *alpha* and McDonald’s *omega* coefficients supported the good internal consistency of both the MMQ total score and its subdimensions. This indicates that even if the items may cover different aspects, each of them mirrors the complexity of the mentalizing construct. Indeed, the subscales are also excellently discriminated from each other. Therefore, although the MMQ factors were shown to be theoretically connected [29], these data also support their distinguishability and the significant informative value that each of them can have both in research and in clinical practice, allowing for the assessment of different forms of imbalances in mentalizing that can characterize distinct disorders in their various manifestations [26,27]. Such findings further confirm the complexity of the construct and support the statistical robustness of the MMQ, for which the cut-offs for both the total and its subdimensions have been provided (see Figure 3).

### 4.2. Network Analysis Approach

Network analysis is a flourishing and functional approach for the clinical context, as it allows to interpret the observed variables of a construct (e.g., symptoms, items, or others based on the data collection method) not as an expression of an underlying or latent factor but as parts of a causal system, i.e., as elements inserted in a complex network in continuous mutual influence and interaction between them (see Borsboom and Cramer [69] for a review).

The first result of this analysis highlighted that most of the medium/high-intensity connections between the nodes outlined relationships between items within their corresponding factor (see Figure 4). Albeit to a lesser extent, edges also emerged between items attributable to different factors: these associations were positive if the subdimensions to which the nodes corresponded were both adaptive (i.e., *Relational Attunement* and *Reflexivity*) or both dysfunctional (i.e., Relational Discomfort and Distrust), and negative if the corresponding subdimensions have been conceptualized as opposite to each other (i.e., *Reflexivity* and *Emotional Dyscontrol*). Therefore, these results seem to corroborate the factor analysis results further, providing converging evidence from different techniques in support of the dimensionality of the MMQ [29]: the questionnaire includes both positive and negative factors that, although distinct, show similarities to each other when they have the same shade (i.e., were both positive or both negative) or were negatively associated when conceptualized as opposites (see Figure 1 and Figure 4). Regarding the centrality indices, item 3 (“*I sometimes experience mood swings I can’t control*”; Emotional Dyscontrol) and, subsequently, other nodes attributable to Reflexivity and Emotional Dyscontrol had the highest *degree* and *closeness* (see Figure 5). Concerning *degree*, these results suggested that the skills in critically thinking, identifying, describing, and managing emotions are the elements that most influence the ability to mentalize, and this is in line with previous research showing higher impairment in mentalizing in alexithymic individuals [70]. Regarding *closeness*, the findings showed that items corresponding to the factors Reflexivity and Emotional Dyscontrol presented the highest amount of short direct and indirect connections with all the nodes in the network. This information could have meaningful applicative implications: since changes in these items can influence the entire network and vice versa, the data of this research suggest the need and usefulness of a clinical focus on increasing the positive dimension of Reflexivity and decreasing the negative factor of Emotional Dyscontrol to favour functional processes of mentalization. This supports some lines of research that highlight that emotional representation skills are the cornerstone to improving mentalizing [71]. Finally, Item 9 (“*Relationships with other people prevent me from being myself*”; Relational discomfort) showed the highest *betweenness*. This indicates that this node has a significant role within the communication flow in the network, acting as a bridge between other elements by being influenced and in turn influencing the other components through mentalizing. Indeed, interpersonal relationships can play a decisive role in the acquisition of mentalizing skills, and, in turn, different components of mentalizing can influence the relational modalities of the individual and his ability to navigate the complex social world [3,72,73,74].

### 4.3. Limitations & Directions for Future Research

In the present research, some limitations should be paid attention to. First, a snowball sampling technique was used to recruit the participants, and this may limit the representativeness of the research sample. Indeed, the second limitation of this study is the gender imbalance, which could be due to the procedure used for data collection: although the size of the sample allows for a significant number of both women and men, the predominance of female participants implies the need to be careful in generalizing the results to men. The recruitment of a more balanced sample through probability sampling procedures may overcome this issue in future research. Finally, a clinical sample was not involved. In fact, since the MMQ does not aim to assess diagnostic components for clinical diseases (although it evaluates a central element for healthy mental functioning [4,11]), a community sample was recruited for this study. However, the use of a clinical sample could be an interesting challenge for future research to evaluate the efficacy of the cut-off scores and to further enrich the results by exploring differences in the dimensions of mentalizing between different clinical and non-clinical conditions. Despite the limitations, this research enriches and completes the previous preliminary results on MMQ [29]. Indeed, the comparison between several alternative factorial models and the use of the HTMT analysis [57] allowed for supporting the psychometric robustness of the scale. Furthermore, the elaboration of the cut-off points widens the possibilities of their use in the clinical and preventive context. Finally, concerning the network analysis, on the one hand, this technique showed coherence with the results of the factorial approach, further validating them; on the other hand, it provided additional information and food for thought for a better understanding of the dynamics between the constituent elements of the mentalization construct, with important applicative outcomes. Therefore, these results support the effectiveness of integrating different approaches to foster useful insights both in the clinical and research fields.

## 5. Conclusions

The MMQ is a self-report questionnaire that adopts a multidimensional lens for assessing mentalizing [29]. Given the complexity of the construct, the present study aimed to deepen the evaluation of the subdimensions investigated by the measure through the integration of different psychometric approaches. The results confirmed the goodness of the psychometric characteristics of the questionnaire, supporting the adequacy of the 6-factor model and highlighting good indications of internal consistency and discriminant validity between the subscales. Therefore, the MMQ has proven to be a valid, theoretically sound, and psychometrically robust measure that can be usefully used both in research and clinical practice. Indeed, given the potential role of imbalances in mentalization as a risk factor for psychopathology [11] or, conversely, of functional levels of mentalization as an element in favour of well-being [9], the research considers the need to consider the different facets of the construct by exploring it in its dimensionality for a better understanding of the specific disorders, with consequent benefits for the tailor-made elaboration of therapeutic interventions. On the other hand, the results of the network analysis also allow offering further insights into the interaction between the constituent elements of the construct, underscoring the central role of the Emotional Dysregulation or, conversely, Reflexivity components in influencing the other dimensions, as well as the potential mediating role of aspects related to Relational Discomfort. Taken together, these results may provide useful information to support targeted and effective clinical and preventive interventions.

## Figures and Tables

**Table 1:** Demographic characteristics of the sample (*N* = 1460).

Characteristics		M ± SD	*N*	%
	Age	33.44 ± 13.284		
*Sex*				
	Males		387	23.6
	Females		1253	76.4
*Marital Status*				
	Single		1077	65.7
	Married		305	18.6
	Cohabiting		195	11.9
	Separated		22	1.3
	Divorced		39	2.4
	Widowed		2	0.1
*Education*				
	Elementary School diploma		2	0.1
	Middle School diploma		91	5.5
	High School diploma		637	38.8
	University degree		510	31.1
	Master’s degree		264	16.1
	Post-lauream specialization		136	8.3
*Occupation*				
	Student		536	32.7
	Working student		274	16.7
	Artisan		34	2.1
	Employee		413	25.2
	Entrepreneur		74	4.5
	Freelance		93	5.7
	Manager		28	1.7
	Trader		40	2.4
	Retired		34	2.1
	Unemployed		114	7.0

**Table 2:** Fit statistics of the MMQ for two factorial models and the chi-square variation test.

	*χ* ^2^	*df*	*p*	NFI	TLI	CFI	RMSEA	SRMR	Models Comparison	Δ*χ*^2^	Δ*df*	*p*
Six-Factor Model	3309.18	477	<0.001	0.90	0.90	0.91	0.06	0.06				
									-	-	-	-
Higher-order Model	3616.64	485	<0.001	0.89	0.89	0.90	0.06	0.08				
									M1–M2	307.46	8	<0.001
Unifactorial Model	14,637.76	492	<0.001	0.54	0.52	0.55	0.13	0.16				
									M1–M3	11,328.58	15	<0.001

***Note***: *χ*^2^ = Chi-square value of model fit; *df* = degree of freedom; NFI = Normed-Fit Index; TFI = Tucker Lewis index; CFI = Comparative Fit Index; RMSEA = the Root Mean Square Error of Approximation; SRMR = Standardized Root Mean Square Residual; M1 = Six-Factor Model; M2 = Higher-order Model; M3 = Unifactorial Model; Δ*χ*^2^ = Difference in *χ*^2^ values between the compared models; Δ*df* = Difference in the number of degrees of freedom between the compared models.

**Table 3:** Indications of internal consistency, MMQ factors’ heterotrait-monotrait ratio of correlations (HTMT), and cut-off points.

Score		*α*	ω	HTMT Analysis					Cut-Off		
				1	2	3	4	5	Low Scores	Average Scores	High Scores
	*MMQ total score*	0.90	0.88						<108	108–132	>132
Positive Factors											
	*1. Reflexivity,*	0.92	0.93	-					<34	34–43	>43
	*2. Ego-strength*	0.88	0.88	0.55	-				<18	18–24	>24
	*3. Relational Attunement*	0.88	0.88	0.83	0.54	-			<17	17–23	>23
Negative factors											
	*4. Relational Discomfort*	0.80	0.80	0.14	0.20	0.09	-		<9	9–15	>15
	*5. Distrust*	0.79	0.80	0.09	0.03	0.10	0.79	-	<8	8–13	>13
	*6. Emotional Dyscontrol*	0.79	0.81	0.30	0.08	0.60	0.32	0.51	<10	10–16	>16

Note: The cut-off points have been calculated by considering the 25th and 75th percentiles for each scale.

**Table 4:** Centrality measures per MMQ item.

	Network				Network				Network		
Item	B	C	S	Item	B	C	S	Item	B	C	S
MMQ1	−1.41	−0.86	−1.21	MMQ12	1.29	−0.06	0.78	MMQ23	1.12	1.71	−0.09
MMQ2	1.97	1.86	1.31	MMQ13	−0.74	−0.82	0.46	MMQ24	−0.68	−0.99	−0.06
MMQ3	0.28	2.03	1.80	MMQ14	−0.90	−0.04	−1.81	MMQ25	−0.79	−0.71	0.63
MMQ4	−1.13	−1.16	−1.24	MMQ15	−0.90	−0.46	−0.50	MMQ26	−0.40	−0.22	−0.37
MMQ5	0.39	−0.74	0.48	MMQ16	0.17	0.40	1.55	MMQ27	−1.58	−1.19	−0.97
MMQ6	0.73	−0.19	1.50	MMQ17	−0.85	−0.22	−1.12	MMQ28	−0.34	−0.58	−0.41
MMQ7	0.73	1.75	1.20	MMQ18	1.01	0.83	0.46	MMQ29	−0.68	−1.09	0.01
MMQ8	0.73	0.42	−0.32	MMQ19	0.56	0.17	−1.66	MMQ30	−1.07	−1.00	0.92
MMQ9	2.14	1.37	1.30	MMQ20	−0.23	−0.39	0.31	MMQ31	1.35	1.55	0.20
MMQ10	−0.57	−0.48	−0.53	MMQ21	0.73	0.13	−0.26	MMQ32	1.07	1.16	0.80
MMQ11	−0.85	−0.56	−1.42	MMQ22	0.00	−0.22	−0.56	MMQ33	−1.13	−1.40	−1.19

Note: B = Betweenness; C = Closeness; S = Strength.

## Data Availability

The data presented in this study are available on request from the corresponding author. The data are not publicly available for privacy reasons.

## Appendix A

MMQ items and scoring instructions can be found here: https://doi.org/10.6084/m9.figshare.21782603.

Alternatively, information can be requested from the corresponding author.
